# Neck ligament strength is decreased following whiplash trauma

**DOI:** 10.1186/1471-2474-7-103

**Published:** 2006-12-21

**Authors:** Yasuhiro Tominaga, Anthony B Ndu, Marcus P Coe, Arnold J Valenson, Paul C Ivancic, Shigeki Ito, Wolfgang Rubin, Manohar M Panjabi

**Affiliations:** 1 Department of Orthopaedic Surgery, St. Marianna University School of Medicine, Kanagawa, Japan; 2 Biomechanics Research Laboratory, Department of Orthopaedics and Rehabilitation, Yale University School of Medicine, New Haven, Connecticut, USA; 3 Department of Orthopaedic Surgery, Dartmouth-Hitchcock Medical Center, Lebanon, New Hampshire, USA; 4 Department of Orthopedic Surgery, Rush University Medical Center, Chicago, Illinois, USA

## Abstract

**Background:**

Previous clinical studies have documented successful neck pain relief in whiplash patients using nerve block and radiofrequency ablation of facet joint afferents, including capsular ligament nerves. No previous study has documented injuries to the neck ligaments as determined by altered dynamic mechanical properties due to whiplash. The goal of the present study was to determine the dynamic mechanical properties of whiplash-exposed human cervical spine ligaments. Additionally, the present data were compared to previously reported control data. The ligaments included the anterior and posterior longitudinal, capsular, and interspinous and supraspinous ligaments, middle-third disc, and ligamentum flavum.

**Methods:**

A total of 98 bone-ligament-bone specimens (C2–C3 to C7-T1) were prepared from six cervical spines following 3.5, 5, 6.5, and 8 g rear impacts and pre- and post-impact flexibility testing. The specimens were elongated to failure at a peak rate of 725 (SD 95) mm/s. Failure force, elongation, and energy absorbed, as well as stiffness were determined. The mechanical properties were statistically compared among ligaments, and to the control data (significance level: P < 0.05; trend: P < 0.1). The average physiological ligament elongation was determined using a mathematical model.

**Results:**

For all whiplash-exposed ligaments, the average failure elongation exceeded the average physiological elongation. The highest average failure force of 204.6 N was observed in the ligamentum flavum, significantly greater than in middle-third disc and interspinous and supraspinous ligaments. The highest average failure elongation of 4.9 mm was observed in the interspinous and supraspinous ligaments, significantly greater than in the anterior longitudinal ligament, middle-third disc, and ligamentum flavum. The average energy absorbed ranged from 0.04 J by the middle-third disc to 0.44 J by the capsular ligament. The ligamentum flavum was the stiffest ligament, while the interspinous and supraspinous ligaments were most flexible. The whiplash-exposed ligaments had significantly lower (P = 0.036) failure force, 149.4 vs. 186.0 N, and a trend (P = 0.078) towards less energy absorption capacity, 308.6 vs. 397.0 J, as compared to the control data.

**Conclusion:**

The present decreases in neck ligament strength due to whiplash provide support for the ligament-injury hypothesis of whiplash syndrome.

## Background

Whiplash injuries leading to chronic symptoms have an estimated annual incidence of one million in the USA [1], resulting in societal costs up to $29 billion [2]. Between 5 and 8% of whiplash patients develop chronic symptoms severe enough to diminish their work capacity [3-5]. Present knowledge remains incomplete regarding the specific anatomical components injured during whiplash and the causes of the resulting chronic symptoms. MRI and autopsy studies have documented cervical ligament, disc, and facet joint injuries in whiplash victims [6-8]. The only clinical evidence comes from Lord et al [9,10], who used nerve block and radiofrequency ablation of facet joint afferents, including capsular ligament nerves, to successfully relieve pain in whiplash patients. To our knowledge, no previous study has documented injuries to the cervical ligaments as determined by altered dynamic mechanical properties due to whiplash.

Simulated rear impacts of six whole cervical spine specimens with muscle force replication and surrogate head produced dynamic ligament strains above physiological limits and mechanical spinal instability [11-14]. The dynamic strains in the anterior longitudinal ligaments [12] and annular fibers [13] above physiological limits were observed at the middle and lower cervical spine. Following whiplash trauma, the ligaments were classified as having sustained no macroscopic rupture, partial rupture with no visible damage to the underlying annulus, or complete rupture with visible anterior annular tears [12]. They found that the ruptured ligaments were associated with significantly greater dynamic intervertebral extension, peak ligament strain, and joint laxity as compared to the uninjured ligaments. In another study, capsular ligaments at C5–C6 and C6–C7 were found to be at risk for subfailure injury due to excessive dynamic strains [14]. The aforementioned results were supported by Ito et al [11] who documented increased joint laxity at the middle and lower cervical spine based upon flexibility tests performed prior to and following each impact. These researchers hypothesized that microscopic subfailure injuries of the cervical ligaments may injure mechanoreceptive and nociceptive nerve endings and lead to pain and chronic symptoms.

Previous *in vivo *animal studies have investigated the effects of excessive capsular ligament tensile strain on resulting injury severity and chronicity [15-18]. Using a rat model, Lee et al [15] measured mechanical allodynia via forepaw withdrawal for up to 7 days following the application of either 11 or 34% capsular ligament strain at C6–C7. The 34% strain resulted in mechanical allodynia and injury that was over three times more severe, as compared to the 11% strain. Using a goat model, Lu et al [16-18] applied tensile load to the C5–C6 capsular ligament and measured the nerve root activity, and capsular ligament load and strain. Correlations between nerve root activity and capsular ligament load and strain indicated that the sensory receptors in the facet joint can detect changes in mechanical stimulus and possibly pain due to non-physiological capsular ligament strain.

While previous studies have reported dynamic cervical ligament strains above physiological limits during simulated whiplash and the effects of excessive capsular ligament strain on resulting injury severity, no study has documented ligament injuries as determined by altered dynamic mechanical properties due to whiplash. We hypothesize that there exist significant differences in the mechanical properties among the cervical ligaments following whiplash, and between the whiplash-exposed and control [19] ligaments. The purpose of this study was to determine the dynamic mechanical properties of the human cervical spine ligaments following whiplash. Additionally, the present data were compared to previously reported control data [19].

## Methods

### Overview

Bone-ligament-bone specimens were prepared from six osteoligamentous whole cervical spine specimens that had been previously rear impacted incrementally at peak T1 horizontal accelerations of 3.5, 5, 6.5 and 8 g [20]. As the incremental trauma and the single trauma produce the same final ligament injury severity [21], the final injury produced was equivalent to that of the 8 g impact. Following the 8 g rear impact, the specimens were frozen at -20°C, prior to preparation for mechanical testing [22]. The ligaments were elongated to failure and the failure force, elongation, and absorbed energy, as well as stiffness were determined. The failure force, elongation, and absorbed energy were compared with previously reported control data [19], obtained using the same methodology as outlined below. Additionally, the failure elongation data were compared with the average physiological ligament elongation, computed using a simple mathematical model. As the present study utilized *in vitro *specimens, no ethical approval was required.

### Specimen preparation

**Figure 1:**
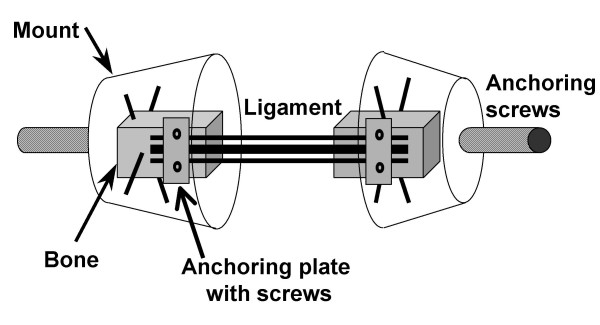
Schematic of a bone-ligament-bone preparation from Ivancic et al [19]. Anchoring plates ensured mid-substance tears during elongation.

The average age of the six whole cervical spine specimens was 70.8 years (range, 52 to 84 years) and there were four male and two female donors. The specimens had no history of any disease that could have affected the osteoligamentous structures. The specimens were divided into two equal groups: the first group was dissected into C2–C3, C4–C5, and C6–C7 functional spinal units (FSUs), while the second group was dissected into C3–C4, C5–C6, and C7-T1 FSUs. Each FSU was sectioned at the pedicles. Anterior elements were sectioned coronally into thirds to create anterior longitudinal ligament (ALL), middle-third intervertebral disc (MTD), and posterior longitudinal ligament (PLL) bone-ligament-bone preparations. Posterior elements were sectioned to create capsular ligament (CL), ligamentum flavum (LF), and intraspinous and supraspinous ligament (ISL+SSL) preparations. Left and right CLs from each intervertebral level were prepared separately, while left and right LFs from each level were prepared as a single unit. Each preparation was then mounted for mechanical testing (Figure 1). To ensure rigid anchoring of bone within quick setting bondo mounts (Evercoat Z-Grip, Fibre Glass-Evercoat, Cincinnati, OH), two perpendicular thru-holes were drilled into each bone in which 19 gauge needles were inserted. Each mount contained an anchoring screw for subsequent attachment to the testing apparatus. To increase fixation of the ALLs and PLLs to the bone, plastic plates were glued atop the ligament attachments and rigidly secured with machine screws. In total, 98 bone-ligament-bone specimens were prepared (Table 1).

### Experimental apparatus

**Figure 2:**
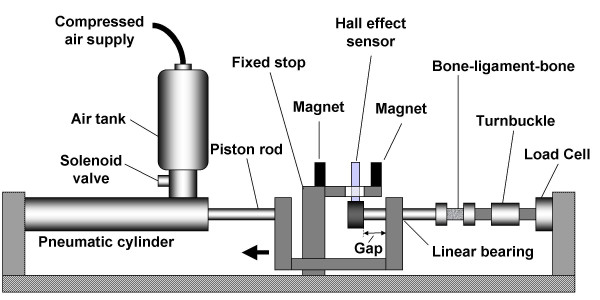
Schematic of the experimental apparatus from Ivancic et al [19]. Air flow controlled via the solenoid valve caused movement of the piston rod, and therefore ligament elongation. Force was measured by a load cell and elongation by a Hall effect sensor positioned between two magnets.

A custom high-speed apparatus was used to elongate the bone-ligament-bone preparations (Figure 2) [19]. The apparatus consisted of a pneumatic cylinder (model 1.5 × 5 Allenair, Minneola, NY) supplied with compressed air via an air tank. Air flow from the tank to the pneumatic cylinder was controlled by a solenoid valve. A controlled gap in the system permitted the pneumatic piston to achieve sufficiently high velocity prior to the onset of ligament elongation. Force was measured with a uni-axial load cell (667 N capacity, model LCCA-150, Omega, Stamford, CT). Elongation was measured using a Hall effect sensor (A3506LU, Allegro Microsystems, Worcester, MA) positioned between two magnets (13 × 13 × 5 mm, part no. PR28ES4187B, Dexter Magnetic, Billerica MA). Immediately prior to testing, the ligament was preloaded to 5 N of tension, and this was defined as zero elongation. The force and elongation data were sampled at 6.3 kHz up to complete ligament rupture. The average (SD) peak elongation rate was 725 (95) mm/s for the whiplash-exposed ligaments and 723 (106) mm/s for the previously reported control [19] ligaments.

### Data analyses

#### Parameters measured

Failure force was defined as the maximum force attained, while failure elongation was the elongation at the failure force. Energy absorbed was calculated by integrating the force between zero and the failure elongation. To obtain ligament stiffness, each force-elongation curve was fitted with a second order polynomial and its derivative evaluated at 25, 50, 75% of the failure force. Average (SD) *r*^2 ^for the fit was 0.97 (0.03).

#### Statistics

To enable statistical testing of the mechanical properties among ligaments, data from all cervical levels (C2–C3 to C7-T1) were combined for each ligament. Single-factor (ligament), non-repeated measures ANOVA (P < 0.05) and Bonferroni post hoc tests were performed to determine differences among ligaments. Adjusted P-values were computed based upon the number of post-hoc tests performed. To enable comparisons of the primary mechanical properties of failure force, elongation, and energy absorbed between the whiplash-exposed and control ligaments [19], data from all ligaments at the spinal levels C3–C4 through C7-T1 were combined within each group. These spinal levels were used as increased ligament laxity has been previously documented at C3–C4 through C7-T1 due to whiplash [11]. Students unpaired t-tests were performed with significance set at P < 0.05 and a trend towards significance at P < 0.1. Adjusted P-values were computed based upon the three statistical tests performed.

### Average physiological ligament elongations

To provide a baseline for the failure elongation data, average physiological ligament elongations were computed using a simple mathematical model. The model consisted of previously reported *in vitro *quantitative anatomy of the cervical spine ligaments [23] and vertebrae [24], average *in vivo *normal intervertebral centers of rotation [25], and average *in vitro *physiological intervertebral rotations [11] specific to each spinal level. The average physiological intervertebral rotation data were obtained using the specimens of the present study prior to simulated whiplash [11]. The upper vertebra was rotated about the center of rotation in flexion and extension to the physiological rotations. The ligament elongations were calculated as the differences in ligament lengths at maximum flexion for PLL, CL, LF, and ISL+SSL and at maximum extension for ALL and MTD, relative to the neutral posture lengths. The data from all spinal levels (C2–C3 to C7-T1) were averaged for each ligament.

## Results

**Figure 3:**
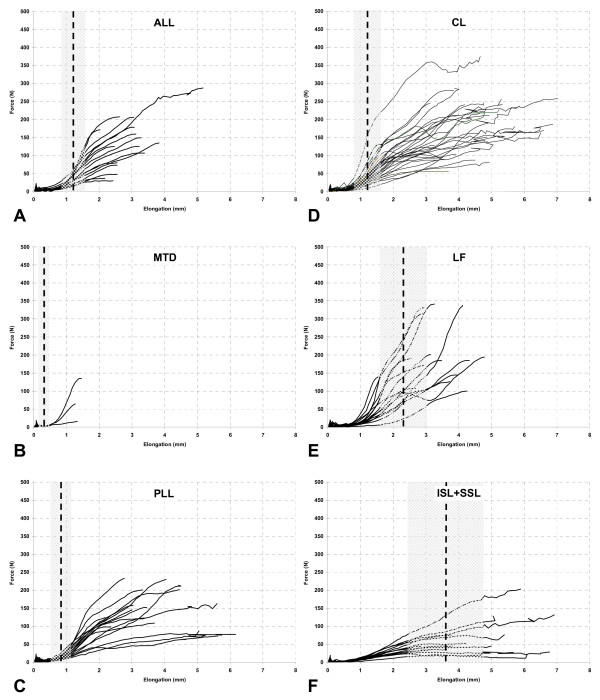
Ligament force vs. elongation curves, including the average physiological elongation shown by a vertical dashed line, and the physiological ligament elongation range (average ± SD), indicated by grey shading. The ligaments included **A) **anterior longitudinal ligament (ALL), **B) **middle-third disc (MTD), **C) **posterior longitudinal ligament (PLL), **D) **capsular ligament (CL), **E) **ligamentum flavum (LF), and **F) **interspinous and supraspinous ligaments (ISL+SSL).

For each ligament, the force-elongation curve, together with the corresponding average physiological elongation and range, are shown in Figures 3A to 3F.

Significant differences in the average failure force, elongation, and energy absorbed were observed among the whiplash-exposed ligaments (Table 2). The highest failure force of 204.6 N was attained in LF, followed by 177.5 N in CL. The failure force in LF was significantly greater than in MTD and ISL+SSL. The highest failure elongation of 4.9 mm was observed in ISL+SSL, followed by 4.5 mm in CL and 3.9 mm in PLL. The failure elongation in ISL+SSL was significantly greater than in ALL, MTD, and LF. The energy absorbed ranged from 0.04 J by MTD to 0.44 J by CL. The energy absorbed by the CL was significantly greater than by the ALL, MTD, and ISL+SSL.

The LF was the stiffest ligament, while ISL+SSL was most flexible (Table 3). At 25% of failure force, the greatest stiffness of 77.2 N/mm was attained in LF, significantly larger than in ISL+SSL. LF stiffness was 96.5 N/mm and 112.1 N/mm at 50 and 75% of failure force, respectively; significantly greater than ALL, PLL, and ISL+SSL.

The average physiological ligament elongations (Table 4), obtained using the mathematical model, ranged from 0.3 mm for MTD to 3.6 mm for ISL+SSL. For all ligaments, the average failure elongation exceeded the average physiological elongation.

In general, the average failure force, elongation, and energy absorbed were lower for the present whiplash-exposed ligaments as compared to the control data [19] (Table 5). The whiplash-exposed ligaments had significantly lower (P = 0.036) failure force, 149.4 vs. 186.0 N, and a trend (P = 0.078) towards less energy absorption capacity, 308.6 vs. 397.0 J.

## Discussion

Whiplash injuries and the causes of the resulting chronic symptoms are not fully understood [6-8]. Percutaneous radiofrequency neurotomy of facet joint afferents in whiplash patients, including the capsular ligament (CL) nerves, is the only clinically documented procedure for pain relief [9,10]. While these clinical studies provide evidence that whiplash may injure the CLs, no previous studies have conclusively determined that the whiplash indeed caused ligament injuries. The present study determined the dynamic mechanical properties of human cervical spine ligaments following whiplash and compared these to control data [19]. The ligaments included the anterior and posterior longitudinal, capsular, and interspinous and supraspinous ligaments, middle-third disc, and ligamentum flavum. Our results indicated that whiplash caused significant decreases in neck ligament strength, as compared to the control data [19] (Table 5). For all whiplash-exposed and control ligaments, the average failure elongation exceeded the average physiological elongation. A few LF (1 whiplash-exposed and 1 control) and ISL+SSL (2 whiplash-exposed and 1 control) specimens [19] failed below the average physiological ligament elongations. As these ligaments may play secondary roles in spinal stability, we believe that the cervical spine would not become unstable if these failures were to occur *in vivo*.

The present study has limitations that must be addressed. The average ages of the whiplash-exposed and control specimens were 70.8 and 80.6 years [19], respectively, due to limited availability of young cadaveric material. Difficulties in specimen preparation and mounting and lack of interspinous and supraspinous ligaments [26] in some spines resulted in decreased sample sizes (Table 1). All specimens that failed via mid-substance tears were included in the data analyses, while ligaments that failed via ligament avulsion from the bone were excluded. Although only six whole cervical spines were available in each of the whiplash-exposed and control groups, a total of 98 and 97 bone-ligament-bone specimens were analyzed in each of the respective groups. In order to determine the effect of whiplash on the primary ligament mechanical properties, data from all ligaments at the spinal levels C3–C4 through C7-T1 were combined within each group. The limited sample sizes precluded determination of statistical differences in the mechanical properties of specific ligaments or spinal levels between the whiplash-exposed and control data [19]. Dynamic ligament elongation along the direction of its fibers was achieved for most ligaments, with the exceptions of the capsular, interspinous, and supraspinous ligaments and middle-third disc, due to anatomical constraints. These ligaments were elongated axially to represent axial separation of a functional spinal unit.

The present study, which has documented altered mechanical properties of cervical spine ligaments due to whiplash, supports previous studies of simulated rear impact, which have reported potentially injurious dynamic ligament strains. Simulated whiplash of cadaveric neck specimens [12] and computational cervical spine models [27] have demonstrated that the average peak anterior longitudinal ligament strains were greatest at the lower cervical spine but were generally below failure thresholds. Panjabi et al [13] documented dynamic annular fiber strains in excess of physiological, with the greatest strains observed at C5–C6. Numerous previous studies have documented potential subfailure injuries to CLs due to excessive dynamic strains [14,28-30]. It has been hypothesized that chronic pain in whiplash patients may be caused by subfailure injuries to the spinal ligaments and embedded mechanoreceptors. Panjabi [31] theorized that corrupted transducer signals from the injured mechanoreceptors may lead to altered muscle response patterns, causing excessive ligament strains and disc and facet loading. These non-physiological load and motion patterns could potentially lead to chronic neck pain via inflammation of spinal nerve roots and ganglia.

Recent *in vivo *animal studies have provided support for the hypothesis that excessive CL strain may cause chronic injury and pain. Lee et al [15] observed greater than three times the injury severity, as measured by mechanical allodynia for up to one week, in a rat model that underwent 34% CL strain at C6–C7, as compared with 11% strain. Mechanical allodynia was measured as an emphatic forepaw withdrawal elicited by a tactile, non-noxious mechanical stimulation to the plantar forepaw surface. The increased mechanical allodynia following the 34% CL strain injury supports the hypothesis that injured mechanoreceptors in the CL causes disturbed muscle response patterns [31]. These findings are supported by the results obtained from a goat model [16-18], in which tensile load was applied to the C5–C6 CL and nerve root activity, and CL load and strain were measured. Correlations between nerve root activity and CL load and strain indicated that nerve activity may be related to the mechanical stimuli. These *in vivo *studies suggest that CL injuries may be the underlying cause of painful chronic symptoms in whiplash patients.

The altered mechanical properties documented in the present study indicate that whiplash loading may cause subfailure injuries to the neck ligaments and embedded mechanoreceptors. These injuries, as documented by decreased ligament strength, may potentially lead to altered facet loading patterns causing excessive synovial fold and facet articular cartilage compression. Synovial fold compression injuries may produce inflammation and pain [32], while articular cartilage injuries can lead to osteoarthritic manifestations [33].

## Conclusion

The present study determined the dynamic failure properties of whiplash-exposed human cervical spine ligaments and compared the results with previously reported control data [19]. Significant decreases in ligament strength were observed following whiplash, supporting the ligament-injury hypothesis of whiplash syndrome. Clinical studies, which have documented pain relief in whiplash patients following nerve block and radiofrequency ablation of facet joint afferents [9,10], provide support for the present results which indicate whiplash loading causes decreased ligament strength.

## Competing interests

The author(s) declare that they have no competing interests.

## Authors' contributions

YT, ABN, MPC, PCI, SI, WR, and MMP contributed to the conception and design of this study and the acquisition of data. AJV, PCI, and MMP performed the data and statistical analyses and drafted the manuscript. All authors have read and approved the final manuscript.

## Pre-publication history

The pre-publication history for this paper can be accessed here:



## Figures and Tables

**Table 1:** Sample sizes for cervical bone-ligament-bone preparations. A total of 98 bone-ligament-bone specimens were analyzed.

	**ALL**	**MTD**	**PLL**	**CL**	**LF**	**ISL+SSL**
**C2–C3**	3	1	3	6	3	3
**C3–C4**	3	1	2	4	3	2
**C4–C5**	3	0	3	6	3	2
**C5–C6**	2	0	3	6	2	3
**C6–C7**	2	0	3	6	2	2
**C7-T1**	3	1	2	6	2	2

**Totals**	**16**	**3**	**16**	**34**	**15**	**14**

ALL: anterior longitudinal ligament, MTD: middle-third disc, PLL: posterior longitudinal ligament, CL: capsular ligament, LF: ligamentum flavum, ISL+SSL: interspinous and supraspinous ligaments.

**Table 2:** Average (SD) failure force (N), elongation (mm), and energy absorbed (J) for whiplash-exposed human cervical ligaments. Mechanical property data were obtained at an average peak elongation rate of 725 (SD 95) mm/s. Significant differences (P < 0.05) among ligaments are indicated in the column *Significant*. A blank entry indicates no significant difference was observed.

	**Failure force**	**Significant**	**Failure elongation**	**Significant**	**Energy absorbed**	**Significant**
			
**ALL**	132.2 (70.1)		3.0 (0.8)	CL,ISL+SSL	0.21 (0.19)	CL
**MTD**	71.6 (59.6)	LF	1.4 (0.1)	PLL,CL,ISL+SSL	0.04 (0.30)	CL
**PLL**	149.4 (54.1)	ISL+SSL	3.9 (1.2)	MTD	0.29 (0.13)	
**CL**	177.5 (73.1)	ISL+SSL	4.5 (1.4)	ALL,MTD,LF	0.44 (0.25)	ALL,MTD,ISL+SSL
**LF**	204.6 (85.1)	MTD,ISL+SSL	3.4 (0.8)	CL,ISL+SSL	0.27 (0.11)	
**ISL+SSL**	67.6 (52.3)	PLL,CL,LF	4.9 (1.3)	ALL,MTD,LF	0.19 (0.16)	CL

ALL: anterior longitudinal ligament, MTD: middle-third disc, PLL: posterior longitudinal ligament, CL: capsular ligament, LF: ligamentum flavum, ISL+SSL: interspinous and supraspinous ligaments.

**Table 3:** Average (SD) stiffness (N/mm) at 25, 50, and 75% of failure force for whiplash-exposed human cervical ligaments. Mechanical property data were obtained at an average peak elongation rate of 725 (SD 95) mm/s. Significant differences (P < 0.05) among ligaments are indicated in the column *Significant*. A blank entry indicates no significant difference was observed.

	**Stiffness at 25%**	**Significant**	**Stiffness at 50%**	**Significant**	**Stiffness at 75%**	**Significant**
			
**ALL**	57.2 (34.5)	ISL+SSL	59.3 (35.5)	LF,ISL+SSL	60.2 (38.5)	LF,ISL+SSL
**MTD**	61.8 (48.9)		85.9 (72.3)	ISL+SSL	104.2 (90.5)	ISL+SSL
**PLL**	55.2 (21.1)	ISL+SSL	55.4 (23.9)	LF	53.8 (30.1)	LF
**CL**	60.7 (29.3)	ISL+SSL	57.6 (28.6)	LF,ISL+SSL	52.0 (32.2)	
**LF**	77.2 (38.8)	ISL+SSL	96.5 (51.0)	ALL,PLL,CL,ISL+SSL	112.1 (61.8)	ALL,PLL,ISL+SSL
**ISL+SSL**	21.7 (10.1)	ALL,PLL,CL,LF	19.1 (9.4)	ALL,MTD,CL,LF	15.7 (9.4)	ALL,MTD,LF

ALL: anterior longitudinal ligament, MTD: middle-third disc, PLL: posterior longitudinal ligament, CL: capsular ligament, LF: ligamentum flavum, ISL+SSL: interspinous and supraspinous ligaments.

**Table 4:** Average (SD) physiological ligament elongations (mm) obtained using the mathematical model.

	**Physiological elongation**
**ALL**	1.2 (0.3)
**MTD**	0.3 (0.1)
**PLL**	0.8 (0.3)
**CL**	1.2 (0.4)
**LF**	2.3 (0.7)
**ISL+SSL**	3.6 (1.2)

ALL: anterior longitudinal ligament, MTD: middle-third disc, PLL: posterior longitudinal ligament, CL: capsular ligament, LF: ligamentum flavum, ISL+SSL: interspinous and supraspinous ligaments.

**Table 5:** Comparison of mechanical properties between whiplash-exposed and control [19] ligaments. Average (SD) failure force (N), elongation (mm), and energy absorbed (J). Data from all ligaments at the spinal levels C3–C4 through C7-T1 were combined within each group. The average (SD) peak ligament elongation rates were 725 (95) mm/s and 723 (106) mm/s for the whiplash-exposed and control [19] ligaments, respectively. Statistical differences are indicated as *P < 0.05 while trends are indicated as ^#^P < 0.1.

	**Control**	**Whiplash-exposed**	**P-value**
**Failure force**	186.0 (107.1)	149.4 (74.0)	0.036*
**Failure elongation**	4.3 (1.8)	4.0 (1.3)	0.702
**Energy absorbed**	397.0 (291.6)	308.6 (201.8)	0.078^#^
